# The Structure of Herpesvirus Fusion Glycoprotein B-Bilayer Complex Reveals the Protein-Membrane and Lateral Protein-Protein Interaction

**DOI:** 10.1016/j.str.2013.05.018

**Published:** 2013-08-06

**Authors:** Ulrike E. Maurer, Tzviya Zeev-Ben-Mordehai, Arun Prasad Pandurangan, Tina M. Cairns, Brian P. Hannah, J. Charles Whitbeck, Roselyn J. Eisenberg, Gary H. Cohen, Maya Topf, Juha T. Huiskonen, Kay Grünewald

**Affiliations:** 1 Department of Molecular Structural Biology, Max Planck Institute of Biochemistry, Martinsried 82152, Germany; 2 Oxford Particle Imaging Centre, Division of Structural Biology, Wellcome Trust Centre for Human Genetics, University of Oxford, Oxford OX3 7BN, UK; 3 Institute of Structural and Molecular Biology, Department of Biological Sciences, Birkbeck College, University of London, London WC1E 7HX, UK; 4 Department of Microbiology, School of Dental Medicine, University of Pennsylvania, Philadelphia, PA 19104-6002, USA; 5 Department of Pathobiology, School of Veterinary Medicine, University of Pennsylvania, Philadelphia, PA 19104-6002, USA

## Abstract

Glycoprotein B (gB) is a key component of the complex herpesvirus fusion machinery. We studied membrane interaction of two gB ectodomain forms and present an electron cryotomography structure of the gB-bilayer complex. The two forms differed in presence or absence of the membrane proximal region (MPR) but showed an overall similar trimeric shape. The presence of the MPR impeded interaction with liposomes. In contrast, the MPR-lacking form interacted efficiently with liposomes. Lateral interaction resulted in coat formation on the membranes. The structure revealed that interaction of gB with membranes was mediated by the fusion loops and limited to the outer membrane leaflet. The observed intrinsic propensity of gB to cluster on membranes indicates an additional role of gB in driving the fusion process forward beyond the transient fusion pore opening and subsequently leading to fusion pore expansion.

## Highlights

• Full-length gB ectodomain has a structure similar to the ectodomain lacking the MPR
• The gB-bilayer structure reveals that the interaction is limited to the outer leaflet
• gB trimers have an intrinsic propensity to interact laterally and form protein arrays
• Arrays of gB trimers on membranes render the fusion pore open state irreversible

## Graphical Abstract

**Figure:**
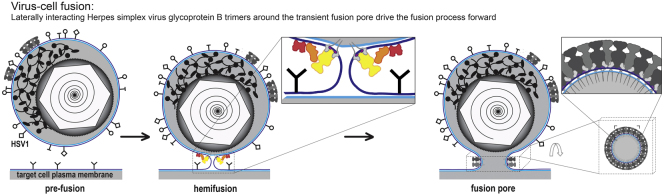

## Teaser

Understanding the mechanism of membrane fusion requires structural information on fusion proteins in the context of the membrane. Maurer et al. solve a structure of herpesvirus gB bound to a membrane. The gB interacts with the membrane at the outer leaflet, which results in a protein coat or belt around the membrane.

## Introduction

Enveloped viruses enter cells by fusing their membrane with that of the host. Dedicated proteins form fusion machinery mediating the merging of the two membranes. The viral fusion machinery can be composed of either a single protein or multiple proteins. In the case of viral fusion, the fusion machinery is primarily located on the virus, in contrast to the case of cell-cell fusion and intracellular fusion, in which the fusion effectors are partitioned between the two membranes (Martens and McMahon, 2008; Sapir et al., 2008; Turner et al., 1998). Regardless of the biological process, the mechanism of membrane merging is believed to be common and to follow the fusion-through-hemifusion pathway (Chernomordik and Kozlov, 2008). For fusion to occur, both membranes need to be in proximity and highly curved. In the case of viral fusion, the fusion protein on the membrane of infectious virus particles is in a metastable state, known as the prefusion conformation (Harrison, 2008). For many fusion proteins, the metastable state follows a priming step usually a result of proteolysis. The metastable state on the infectious virion is restrained by either an intramolecular covalent peptide (e.g., the case of hemagglutinin) or intermolecular interaction (e.g., the case of Dengue prM-E). Upon activation that can be triggered by low pH, endosomal environment or protein-protein interaction with either a coreceptor in *trans* or a member of the complex fusion machinery in *cis* (i.e., on the same membrane side as the fusion mediating protein), hydrophobic fusion loops (FLs) are getting exposed and the protein adopts an intermediate extended conformation to reach the target membrane (Harrison, 2008). This transient extended intermediate conformation then collapses, which brings the two membranes into proximity and, at the same time, induces curvature (Graham and Kozlov, 2010). The high curvature and proximity prime the bilayers for hemifusion that is followed by formation of a transient fusion pore that may flicker between open and closed. It has been suggested that a final conformational change of the fusion protein renders the open state irreversible (Harrison, 2008). Ultimately, in the final stage of fusion, the nascent fusion pore is been expanded to allow for full content mixing.

Glycoprotein B (gB) of herpes simplex virus type 1 (HSV-1), the prototype species of the alphaherpesviruses, is essential for virus entry (Turner et al., 1998). However, gB alone is not sufficient for entry, but is part of the herpesvirus multicomponent fusion machinery (Cai et al., 1988; Campadelli-Fiume et al., 2012; Forrester et al., 1992; Heldwein and Krummenacher, 2008; Ligas and Johnson, 1988; Rey, 2006; Roop et al., 1993). This machinery further includes the receptor binding glycoprotein D (gD) (Montgomery et al., 1996) and the heterodimer of glycoprotein H and L (gH/L) assigned to be the fusion regulator (Chowdary et al., 2010).

The infectious virus particles of HSV-1 display approximately ten different glycoproteins on the virus membrane (Steven and Spear, 1997). Earlier electron microscopy of negatively stained HSV-1 virions has shown protein spikes of various shapes on the viral envelope membrane including characteristic long spikes (Stannard et al., 1987). These long spikes have been identified as gB by immunolabelling (Stannard et al., 1987). The diversity in spike shapes was also evident in cryo-electron microscopy (cryo-EM) and cryo-electron tomography (cryo-ET) on isolated HSV-1 virions (Grünewald et al., 2003) and during virus entry (Maurer et al., 2008).

More recently, the crystal structures of the HSV-1 gB ectodomain, at both neutral and acidic pH, and that of gB of the Epstein Barr virus (EBV) have been determined (Backovic et al., 2009; Heldwein et al., 2006; Stampfer et al., 2010). These structures are highly similar to each other, showing elongated homotrimers ∼16 nm high and ∼8 nm wide. Three discrete regions can be assigned to the homotrimer along its long axis (Figure S1 available online). At one end is the “crown” region formed by domains *IV* of the three protomers. At the other end is the “base” region formed by the three domains *I* that contain the fusion peptides, and the three domains *V*. Based on this domain organization, gB was assigned to the family of class III fusion proteins; other members include glycoprotein G from vesicular stomatitis virus (VSV G) and the baculovirus major envelope glycoprotein gp64 (Figure S1A; Backovic and Jardetzky, 2009; Heldwein et al., 2006; Kadlec et al., 2008; Roche et al., 2006). In all of those three cases, the structure revealed an elongated hairpin conformation and thus indicated postfusion conformation. For VSV G in addition to the low pH structure, an additional structure has been determined at natural pH revealing a more compact prefusion conformation (Roche et al., 2007). Intriguingly, the FLs in both the pre- and the postfusion conformation of VSV G are situated at the same side as the C terminus leading to the transmembrane region. Modeling the membrane-anchored VSV G suggested that the FLs are close to and facing the viral membrane in both forms (Harrison, 2008; Roche et al., 2007, 2008). This modeling raised the question: what prevents the self-insertion of the FLs into the viral membrane?

All class III fusion proteins have a membrane proximal region (MPR) at the C terminus of the ectodomain (Figure S1A; Table S1; Backovic and Jardetzky, 2009). The MPR is not essential for folding and trimerization of the ectodomain, as demonstrated by crystal structures of the ectodomains lacking the MPR (Backovic et al., 2009; Heldwein et al., 2006; Kadlec et al., 2008; Roche et al., 2006, 2007; Stampfer et al., 2010). However, the MPR was shown to be important for virus infectivity and fusion (Jeetendra et al., 2003; Li and Blissard, 2009; Wanas et al., 1999). The structure of the MPR remains unknown for any of the class III fusion proteins, but based on the model of the membrane-anchored VSV G, it seems likely to be in proximity to the FLs (Harrison, 2008; Roche et al., 2007, 2008).

The crystal structures of truncated ectodomains for all HSV-1 fusion machinery components are available. However, understanding the mechanism of membrane fusion at the molecular level requires studying structures of fusion proteins in the context of the membrane. To this end, we determined the structure of gB bound to liposomes with cryo-ET subtomogram averaging. We found that the gB ectodomain lacking the MPR interacted efficiently with liposomes and laterally, resulting in coat formation of postfusion gB on membranes. The presented gB-bilayer complex structure revealed unequivocally that binding is mediated by interaction of the protein base region with the membrane outer leaflet.

## Results

### Clusters of Elongated Glycoprotein Spikes on Native HSV-1 Virions

**Figure 1:**
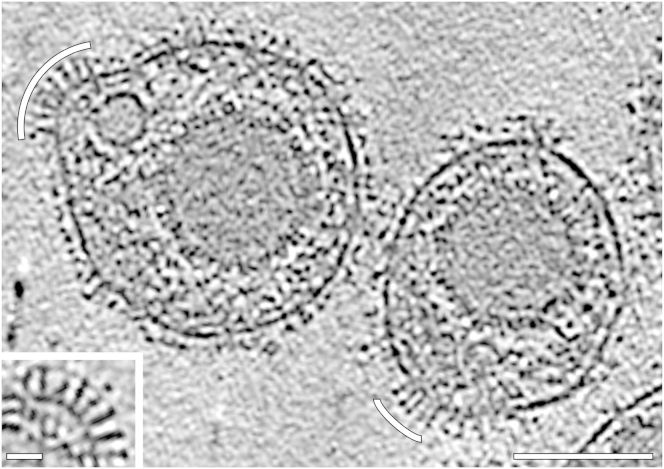
Clusters of Elongated Protein Spikes on Native HSV-1 Virions Slice through a tomogram reconstructed from a cryo-ET tilt series collected at 300 keV and −4 μm defocus. White arcs indicate clusters of elongated spikes reminiscent of gB. Scale bar, 100 nm. Inset shows enlargement of such a cluster. Scale bar, 20 nm. See also Figure S3.

We revisited infectious virions using cryo-ET, applying imaging conditions more advanced than those used earlier (Grünewald et al., 2003; Maurer et al., 2008; see Experimental Procedures). Elongated spikes, reminiscent of the overall shape of the postfusion crystal structure of gB, were observed on the virus envelope (Figure 1). Characteristic distinct densities along the long axes were recognizable for each spike. These elongated spikes appeared often in clusters on the virions and their orientations were uniform with the “crown” regions most likely positioned distal and the “base” proximal to the membrane (Figure 1). This orientation supports previous epitope mapping results (Bender et al., 2007; Hannah et al., 2009), suggesting that domain *IV* is exposed on the virus (Heldwein et al., 2006). This orientation implies that the FLs are in proximity to the viral membrane.

### Comparison of the Ultrastructure of the Full-Length gB Ectodomain with Ectodomain Lacking the MPR

**Figure 2:**
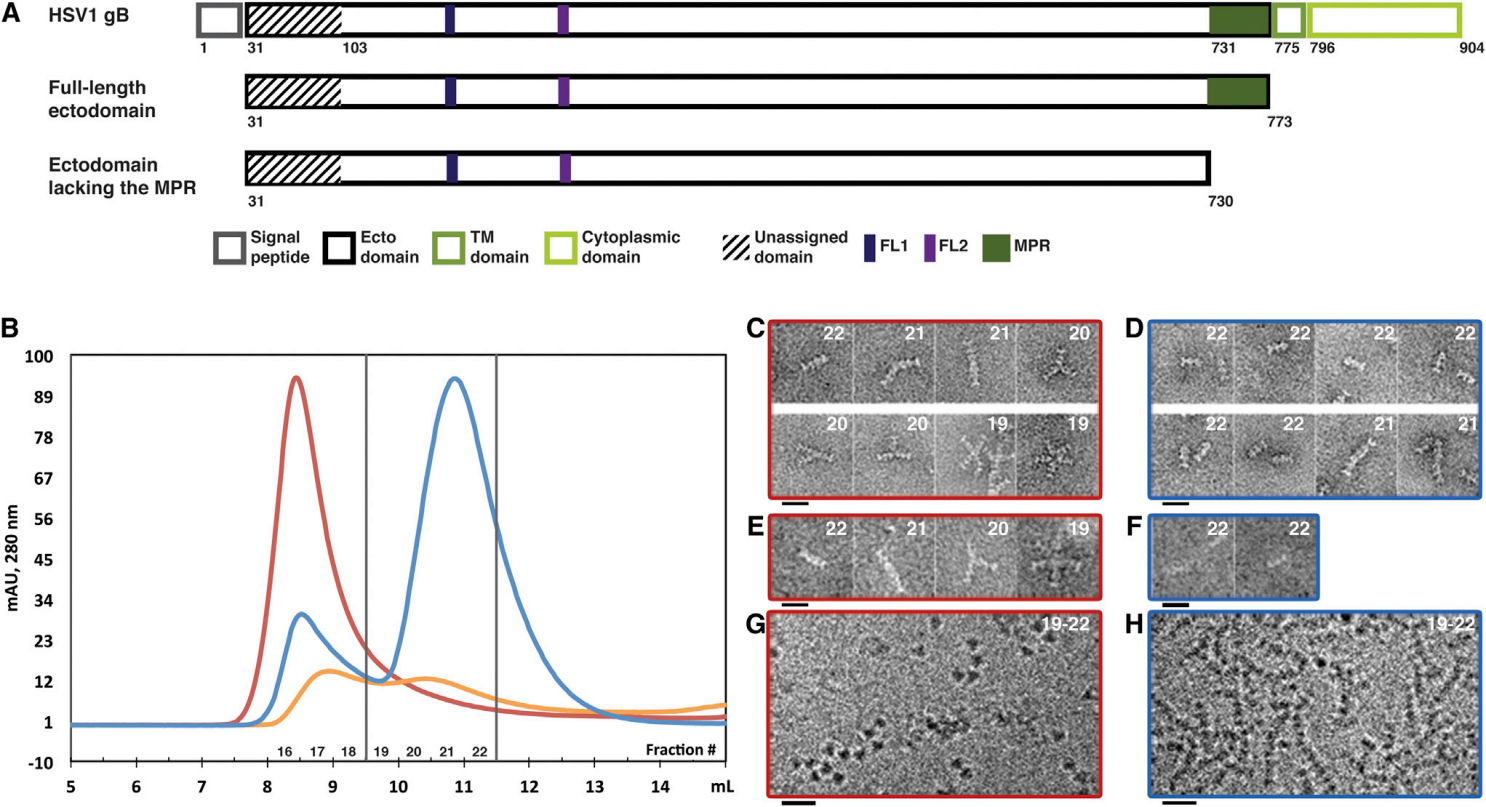
The Full-Length gB Ectodomain and the Ectodomain Lacking the MPR Form Trimers of Overall Similar Shape (A) Schematic illustration of the general topology of HSV-1 gB and the ectodomain constructs used. TM, transmembrane; FL, fusion loop; MPR, membrane proximal region. Numbers below give amino acids at begin, and respectively end, of the domains. (B) Overlay of the SEC of the full-length ectodomain in 2× PBS (red line) or in 2× PBS/0.5% octyl-glucoside (orange line) and the ectodomain lacking the MPR in 2× PBS (blue line). (C–H) Projection EM images of SEC fractions eluted in the absence of detergent (fraction number indicated at the upper right corner) of the full-length gB ectodomain (C, E, and G: red frame) and of the ectodomain lacking the MPR (D, F, and H: blue frame). (C–F) Micrographs of negatively stained particles at pH 5.5 (C and D) and at pH 8.0 (E and F). (G and H) Cryo-EM projection images. Full-length ectodomain particles embedded in vitreous ice were found preferentially oriented with their 3-fold axis perpendicular to the EM grid and thus observed as triangles (G). Scale bars, 20 nm. See also Table S1 and Figures S1 and S2.

To study the ultrastructure of the full-length ectodomain of gB and to compare it to the ectodomain lacking the MPR, they were analyzed using negative stain EM and cryo-EM projection images (Figure 2). The two forms of the gB ectodomain (Figure 2A) were overexpressed in a baculovirus expression system as secreted proteins and purified by immunoaffinity chromatography from the medium. Each of the two forms was further fractionated by size exclusion chromatography (SEC) based on molecular weight (MW) and shape (Figure 2B). For the ectodomain lacking the MPR (Figure 2B, blue curve), a major peak was observed in SEC eluting at ∼11 ml corresponding to the MW of a trimer and a minor peak at the column void volume (∼8.5 ml), which corresponds to soluble protein aggregate. For the full-length ectodomain (Figure 2B, red curve), one major peak was observed at the column void volume with a pronounced “tail”. When a similar amount of the full-length ectodomain was treated with 0.5% octyl-glucoside and run on SEC in the presence of 0.5% octyl-glucoside (Figure 2B, orange curve), two peaks were resolved. Relative to the run without detergent (red curve), the absorbance of the void decreased by ∼10-fold while the absorbance at ∼10.5 ml increased less than 2-fold. This indicated that the detergent dissociated the larger aggregates and that the absorbance in the void volume was boosted artificially due to light scattering. SDS-PAGE confirmed that the relative amounts of protein in the fractions of the void volume for the two SEC runs for the full-length ectodomain (orange and red curves) were comparable (Figure S2).

Fractions 19–22 (marked in Figure 2B) from the runs of the protein in the absence of detergent were further analyzed with cryo-EM (Figures 2G and 2H) and negative stain EM at either pH 5.5 (Figures 2C and 2D) or pH 8 (Figures 2E and 2F) to rule out any pH-induced conformational changes induced by low pH. In fraction 22, for either of the ectodomain forms, elongated particles were observed with the full-length ectodomain particles (Figures 2C and 2E) being hardly distinguishable from the ones for the ectodomain lacking the MPR (Figures 2D and 2F). The overall shape of those particles is highly similar to the postfusion crystal structure of the trimers (Heldwein et al., 2006; Stampfer et al., 2010). This confirmed that fraction 22 indeed contained trimers for both the full-length ectodomain and the ectodomain lacking the MPR. Moreover, particles in fraction 22 also seemed to be trimers under more native conditions when embedded in vitreous ice and imaged with cryo-EM (Figures 2G and 2H). For the full-length ectodomain, the protein particles were observed as 3-fold symmetric densities of ∼8 nm diameter (Figure 2G). The diameter of the particles corresponded well with the trimer cross-section of the crystal structure. The full-length ectodomain showed a preferred orientation in the vitreous ice whereas the ectodomain lacking the MPR did not show a preferred orientation (compare Figures 2G and 2H).

Dimers of trimers were observed in fraction 21 for both ectodomain forms but at substantially lower frequency for gB lacking the MPR (Figures 2C–2F). For the full-length ectodomain, trimers of trimers were observed in fraction 20 and tetramers of trimers and pentamers of trimers were observed in fraction 19 (Figures 2C and 2E); for the ectodomain lacking the MPR, fractions 19 and 20 were not analyzed with negative stain. Despite the heterogeneity in terms of the number of trimers of the full-length ectodomain, trimer self-association seemed to be always mediated by the base region, where the MPR is located at the ectodomain C terminus (Figures 2C and 2E). Previously, it was reported that the highly hydrophobic fusion peptides of EBV-gB induced association of its ectodomain trimers lacking the MPR to form similar supramolecular assemblies (Backovic et al., 2007). However, based on our SEC analysis, for gB of HSV-1 the ectodomain trimers formed supramolecular assemblies predominantly when the hydrophobic MPR was present (Figures 2C and 2E).

### Only the gB Trimers Lacking the MPR Interacted Specifically with Liposomes

**Figure 3:**
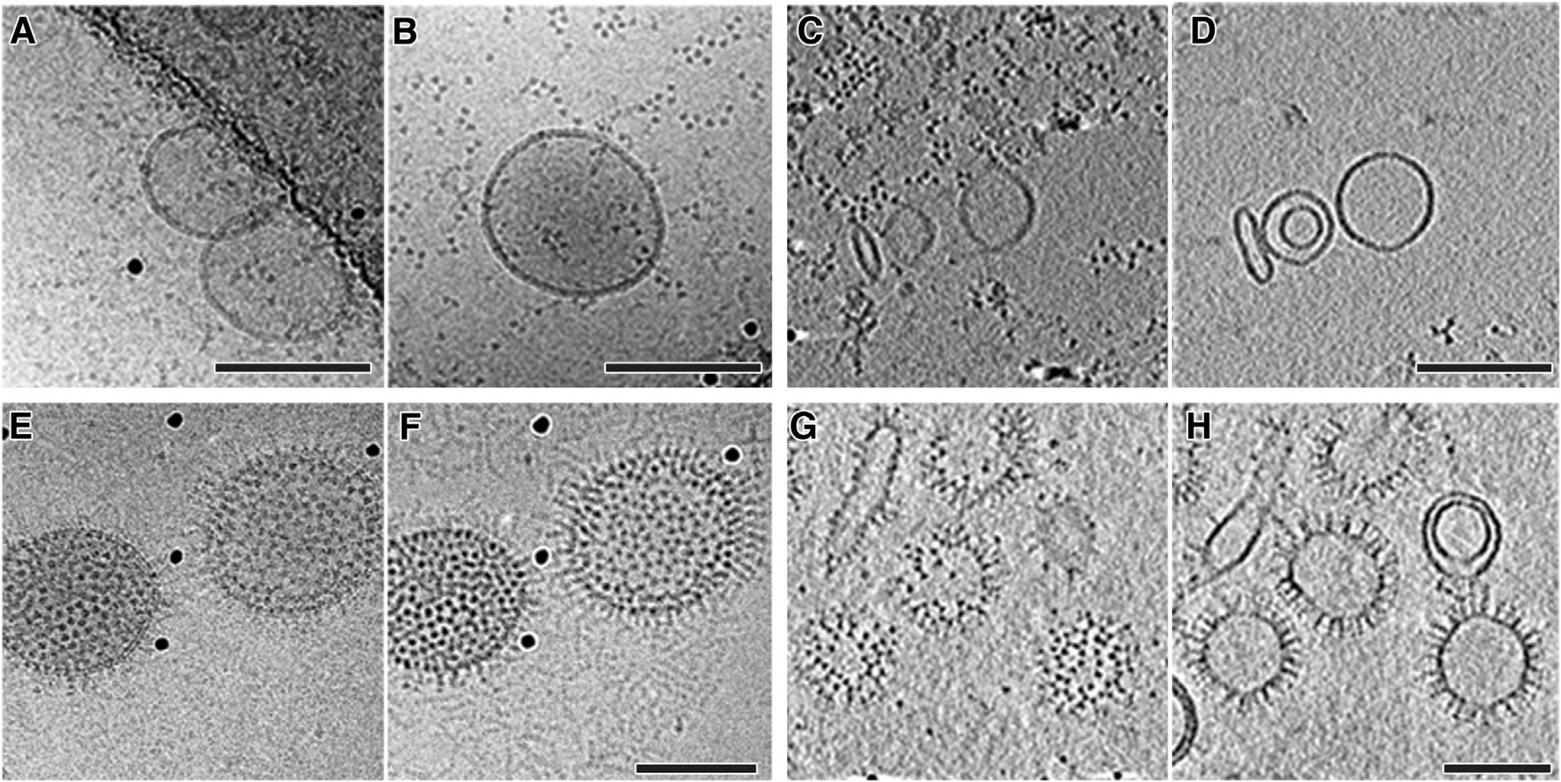
The Full-Length Ectodomain Trimers of gB Failed to Interact with Liposomes while the gB Ectodomain Trimers Lacking the MPR Interacted Specifically with Liposomes and Formed a Protein Coat (A–H) Full-length ectodomain trimers (A–D) and ectodomain trimers lacking the MPR (E–H) were incubated with liposomes before vitrification and cryo-EM imaging. (A, B, E, and F) Cryo-EM projection images, collected at 300 keV at two different defoci, optimized for either visualization of membrane leaflets (A and E: −2 μm defocus) or protein densities (B and F: −6 μm defocus). (C, D, G, and H) Cryo-ET computational slices oriented tangential (C and G) and central (D and H) through the liposomes. Full-length ectodomain trimers (A–D) are observed as triangular densities in the background not interacting with the liposomes that remain bald. In contrast, gB ectodomain trimers lacking the MPR (E–H) were densely decorating the liposomes; excess of protein can be seen in the background (F). Black spherical densities are gold fiducial markers. Scale bars, 100 nm. See also Figures S1 and S3.

To study the propensity of the ectodomain of gB to interact with membranes, the trimeric fraction from the SEC run without detergent, namely fraction 22, of each of the two ectodomain constructs was incubated with liposomes. For the liposome-interaction experiments, the trimeric fractions for each of the ectodomain form were concentrated to 2 mg/ml (in the absence of detergent and no aggregation was observed) before incubation with liposomes. The full-length ectodomain trimers failed to interact with liposomes and the protein particles were observed as triangular trimers in the background of the liposomes in cryo-EM projection images (Figures 3A and 3B) and close to the air-water interface in cryo-ET slices (Figure 3C).

Ectodomain trimers of gB lacking the MPR interacted efficiently with liposomes and formed a coat-like layer (Figures 3E–3H). This interaction was shown not to be of an electrostatic nature because it was resistant to high salt concentrations (see Experimental Procedures; Figure S3A). Moreover, gB bound in a defined orientation, with each individual protein trimer protruding radially outward, appearing as spikes on the membrane (Figures 3E–3H). The binding was cholesterol independent as well as pH independent over the studied range of pH 7.4 (Figures 3E and 3F) to 5.5 (Figures 3G and 3H).

### The Base Region Mediates the Interaction with the Outer Leaflet of the Lipid Bilayer

**Figure 4:**
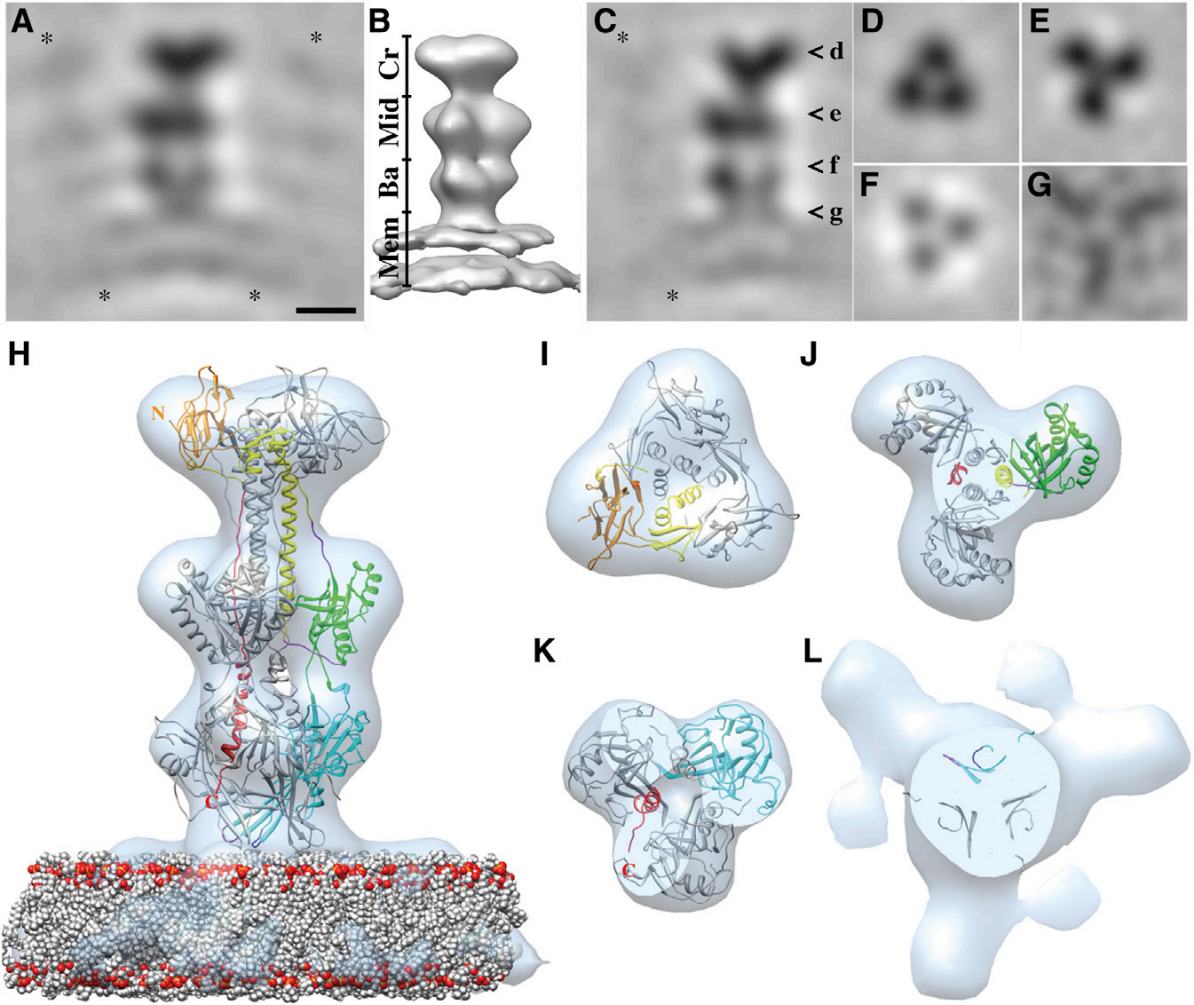
Structure of the gB-Lipid Bilayer Complex (A and B) Side views of the resultant EM density map of the gB-lipid bilayer complex when the emerging angle from the membrane was kept fixed (see Experimental Procedures): central slice through the density map (A) and isosurface rendering (B). The main protein regions (Cr, crown; Mi, middle; Ba, base) and the leaflets of the membrane (Mem) are marked. (C–G) The final EM density map; side view (C) and slices orthogonal to this view (D–G). The respective distances of the slices from the membrane are indicated by open arrowheads in (C). The gray scale in (G) is optimized to highlight the features. Scale bar for (A)–(G), 50 Å. Asterisks indicate neighboring spikes. (H–L) Side view and corresponding orthogonal views (at distance to the membrane similar to D–G) of the pseudo-atomic model (protein, ribbon representation; lipid bilayer, sphere representation) of gB-lipid bilayer complex on the final cryo-ET-derived density map (light blue). One protomer colored according to the domain boundaries and FL 1 and 2 in magenta and dark blue, respectively (Heldwein et al., 2006; Figure S1B); phosphate head groups in red, and choline and carbon tail in gray. N and C indicate the N and C termini. See also Figures S1, S4, and S5 and Movie S1.

To study the nature of the gB-membrane interaction, the structure of the gB-bilayer complex within the coat was determined at pH 5.5 (Figures 4A–4G). This pH was chosen because the FLs in the low pH crystal structure seemed more exposed and thus possibly in a conformation more relevant for membrane insertion. Several hundreds of cryo-ET subvolumes, each with a gB spike in the center, were iteratively aligned and averaged (see Experimental Procedures; Figure S4). Three-fold symmetry was apparent for the protein part already in the early stages of structure refinement (Figure S4F). To guide the alignment, we first assumed that the gB trimers extended radially from the membrane, i.e., the emerging angle from the membrane was kept fixed. This allowed the structure of the trimer to be determined to 3.0-nm resolution (Figures 4A and 4B). In the final refinement stage, gB trimers were allowed to tilt relative to the membrane, resulting in an improvement in resolution to 2.7 nm (Figures 4C–4G; Figure S4G). It is apparent from the structure of the gB-bilayer complex that the protein binds to the target membrane as a trimer via the base region (Figures 4A–4G). The two leaflets of the bilayer were readily resolved (Figures 4A–4C). No density connecting the two leaflets of the lipid bilayer was observed consistent with the interaction being limited to the outer leaflet. Densities of neighboring spikes were evident (Figures 4A and 4C), suggesting lateral interactions of the spikes within the protein coat. At the protein-membrane interface, where the gB base interacts with the lipid bilayer, a tripod-shaped density with its legs at a shallow angle to the membrane was evident (Figure 4G).

The crystal structure of the gB trimer as well as a lipid bilayer were initially each fitted as one rigid body into the final cryo-ET density map (Figures 4H–4L). For the protein part, the three distinct regions of gB—crown, middle, and base—that could be readily identified facilitated the fitting, and a good initial overall fit was obtained for both available crystal structures, viz. neutral and low pH forms (Heldwein et al., 2006; Stampfer et al., 2010; see Experimental Procedures; Figure S5A). The hydrophobic residues of the FLs were not exposed in either of the crystal structures; therefore, their conformation is not optimal for membrane interaction. To improve the fitting of the base region, consisting of domain *I* with its two putative FLs, it was further divided to tertiary-structure elements (Figure S5B). Fitting of these elements resulted in the three domains *I* moving closer to the membrane, separating from each other and orienting the FLs toward the tripod legs (Figure 4L; Figures S5E–5I; Movie S1). It is clear from this fitting (Figures 4H–4L) that the binding of gB to the membrane is mediated by the interaction of the FLs region with the outer leaflet of the membrane (cf. protein-lipid interface shown in Figure 4L). Interestingly, the conformation of FL 2 of the neutral pH crystal structure fitted better to the map (see Experimental Procedures).

### Lateral Protein-Protein Interaction in the Protein Coat on the Liposome Membrane

**Figure 5:**
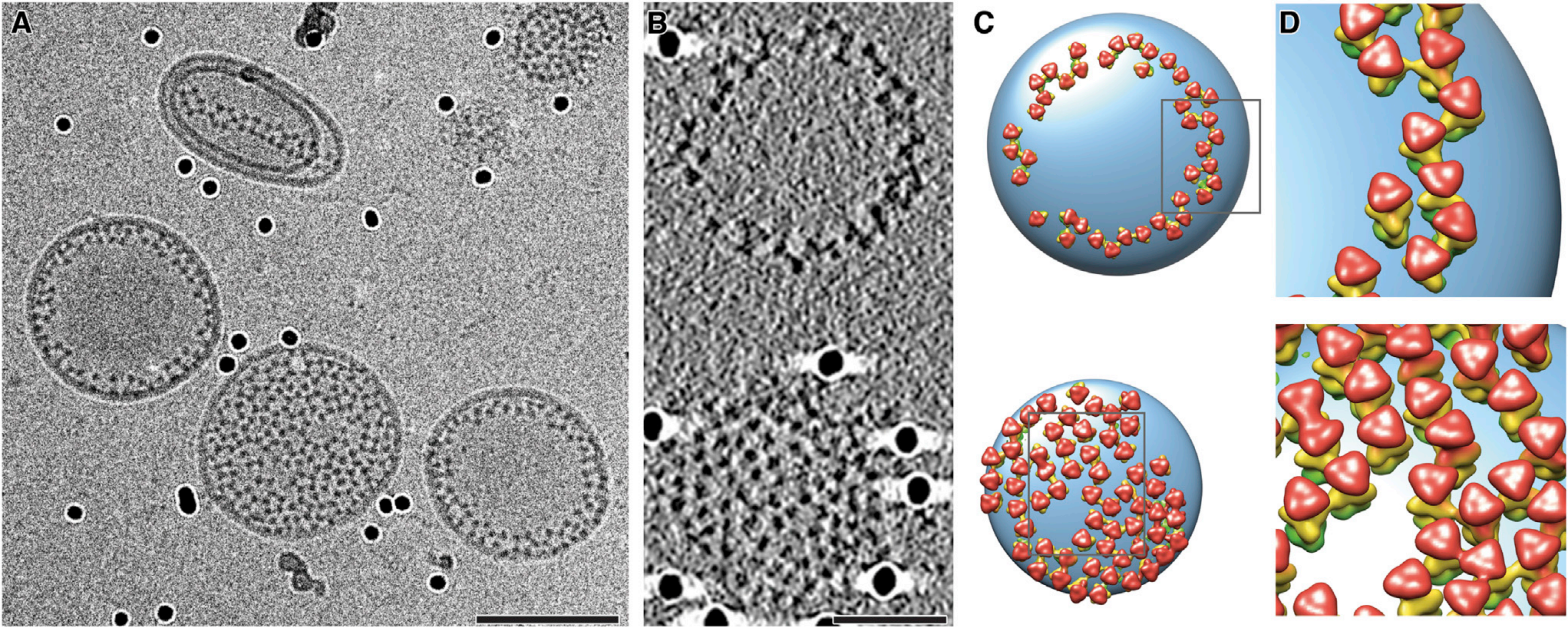
Lateral Interaction of gB Trimers Induced Protein Coat or Belt Formation on Liposomes. With an excess of liposome, the trimers lacking the MPR formed typical two-spike-wide belts or coats on the membrane. (A) Cryo-EM projection image. (B) Cryo-ET slice. Dark spherical densities are gold fiducial markers. (C) Three-dimensional model of the liposomes shown in (B). The EM density map of the gB-lipid bilayer complex was placed in the experimentally determined spike orientations. (D) Zooms into the areas marked in (C) and rotated by 30° around the horizontal axis. Protein domain colors are crown, red; middle, yellow; and base, green. Membrane is light blue. Scale bars for (A), 100 nm; for (B), 50 nm.

To probe whether the observed lateral interaction of spikes was specific or an effect of the high protein concentrations that were used, we repeated the experiment with an excess of liposomes relative to protein. Under those conditions, gB trimers lacking the MPR still interacted with liposomes leading to partially covered liposomes (Figure 5). Notably, individual trimers were not observed. Trimers were either part of a larger coat or part of a two-spike-wide belt-like arrangement. Within both arrangements, trimers were tightly packed and arranged at a minimal center-to-center distance of ∼8 nm (Figures 5C and 5D). To analyze the nature of the lateral interactions between gB trimers, for all individual spikes, the EM structure (Figure 4) was placed on the liposome in the determined trimer positions (Figure 5C). Between trimers, the pairwise relative angular orientation (around the trimer long axis) showed a preference for a rotation of 60° to each other with the middle regions contacting each other (Figure 5D).

## Discussion

Entry of HSV-1 into the host cell depends on the concerted action of a complex fusion machinery involving four glycoproteins (Cai et al., 1988; Forrester et al., 1992; Heldwein and Krummenacher, 2008; Ligas and Johnson, 1988; Rey, 2006; Roop et al., 1993). It is hypothesized that interaction of gD with a cell receptor initiates a cascade of activations of the fusion machinery components: the activated form of gD activates gH/gL that then triggers activation of gB (Atanasiu et al., 2010; Chowdary et al., 2010).

The interaction of hydrophobic peptides of the fusion protein with the target membrane is believed to be an essential step for curving the membrane, which primes the bilayers for hemifusion (Campelo et al., 2008). We have shown that the ectodomain of gB lacking the MPR interacted very efficiently with membranes. The interaction was limited to the outer leaflet of the membrane bilayer and the base region mediated this interaction. The interaction of gB with the membrane did not induce a major conformational change compared to the crystal structures consistent with the conformation being postfusion. In the crystal structures, the hydrophobic residues of the FLs are not exposed, thus it appears that their conformation is suboptimal for membrane interaction. Therefore, to facilitate the interaction, local conformational changes leading to exposure of the hydrophobic residues are expected to occur. Fitting of tertiary-structure elements of the base region into our cryo-ET density map resulted in subtle conformation changes in this region. The resolution of the gB-lipid bilayer complex presented here does not allow fitting of individual secondary structure elements, however, it is plausible that if the secondary structure elements of domain *I* were allowed to move independently during fitting, the beta strands leading to the FLs might have moved even further into the leg densities. Such shallow angle movement would lead to the formation of an extended surface for interaction between protein side chains and the outer membrane leaflet, namely forming a “fusion patch.” This implies that residues from the beta strands leading to the FLs might be also involved in the protein-lipid interaction. Together, the charged and hydrophobic residues of such a “fusion patch” can anchor the protein on the membrane by interacting with the charged lipid head groups and hydrophilic carbon tails, respectively. For VSV G, the prototype class III fusion protein, it was suggested that an arginine restricts the depth of insertion of the hydrophobic FLs (Roche et al., 2006). The underlying basis for the interaction of gB with the membrane is most likely similar, the major difference being the interaction angle with the membrane, i.e. perpendicular for VSV G and shallow for gB.

We observed that gB trimers in the postfusion conformation laterally interacted on the membrane and formed a protein coat or belt around the membrane. Notably, the full-length ectodomain showed a preferred orientation in the vitreous ice and the distribution was not completely random (Figure 2G). The hydrophobic air-water interfaces of the thin aqueous film on the cryo-EM grid (Taylor and Glaeser, 2008) oriented the particles perpendicular to the EM grid (Figure 2G). Lateral protein-protein interaction then induced local arrangements into groups of trimers. Moreover, lateral assembly of full-length gB was also observed on virions (Figures 1 and S3B). The laterally interacting gB on virions can be interpreted as belonging to a fraction of gB that was prematurely triggered into its postfusion form. The presence of both pre- and postfusion forms of the fusion protein on the viral membrane had been previously reported for parainfluenza virus in cryo-negative-stained specimens (Ludwig et al., 2008). The importance of the observation of the assembly of gB on the viral membrane is to show that the ability to laterally interact is an intrinsic property of gB and not only of the recombinant ectodomain. Similarly, postfusion trimers forming a lattice on liposomes were reported for a class II viral fusion protein (Gibbons et al., 2004).

It was proposed that lateral assembly of fusion proteins into an interconnected protein ring around a transient fusion pore drives the fusion reaction forward to an irreversible state and for pore expansion (Chernomordik and Kozlov, 2008; Harrison, 2008; Leikina et al., 2004). The ability of the postfusion conformation of gB to laterally interact on membranes, as observed here, then indicates an additional role for gB in stabilizing the transient fusion pore. Such a role is in agreement with the observation that gB is required for content mixing (Subramanian and Geraghty, 2007) and is in addition to its role in the early stages of the fusion reaction (Jackson and Longnecker, 2010).

**Figure 6:**
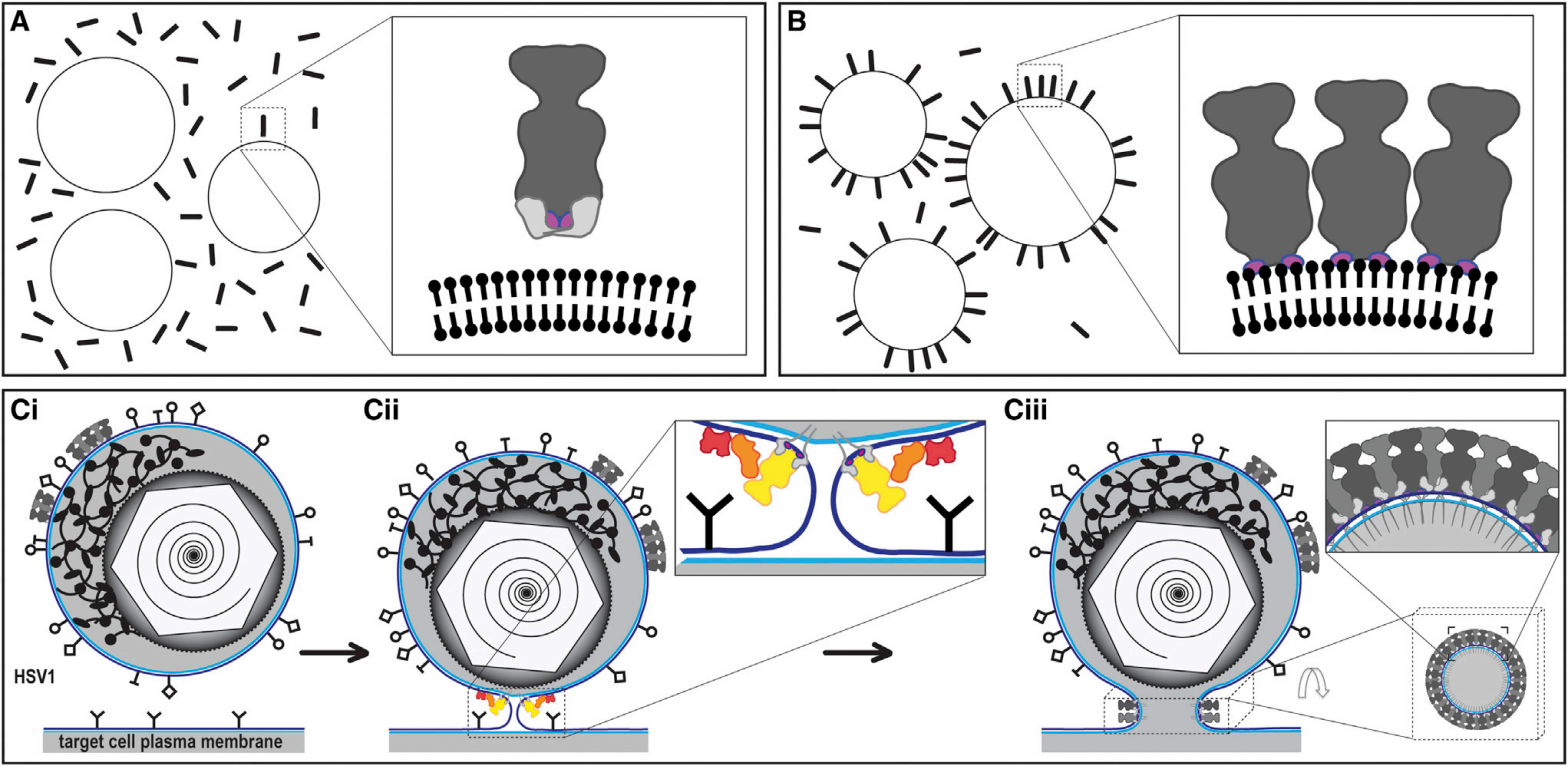
gB-Membrane Interaction and Its Implications (A) The full-length ectodomain of gB (sticks) does not interact with liposomes (circles). Inset shows zoom into area marked on the left schematic drawing. The MPR is light gray. (B) Ectodomain of gB (sticks) lacking the MPR is capable of interacting with liposomes (circles). Inset shows zoom into area marked on the left schematic drawing. In the absence of the MPR, the FLs (colored pink/purple) mediate efficient interaction with liposomes, which is limited to the outer leaflet of the membrane, and laterally between gB trimers. (C) Model of gB function in fusion pore expansion. (i) Virion of HSV-1 (top, containing icosahedral capsid) before interaction with the target cell (bottom). (ii) Hemifusion intermediate state with the activated multicomponent fusion machinery (enlarged in inset) composed of gD (red), gH/L (orange), and gB (yellow). Outer membrane leaflet, dark blue; inner membrane leaflet light blue. (iii) Clustering of postfusion gB trimers around the fused membrane of the transient fusion pore render the fusion pore open state irreversible. Bottom right: orthogonal view of the cuboid indicated by the dashed box. Upper right: zoom into the region indicated by corners in bottom right.

In summary, we observed that the MPR impedes interaction of the gB ectodomain with liposomes (Figure 6A). Experimental removal of the MPR leads to efficient interaction of gB ectodomain with liposome membranes (Figure 6B). The structure of the gB-bilayer complex presented here reveals that the interaction is with the outer membrane leaflet only. Lateral interaction of gB trimers on the membrane drives the formation of a protein belt. Such assembly around the neck of a transient fusion pore (Figure 6C) renders the open state irreversible. This might also be the driving force facilitating pore expansion and ultimately leading to HSV-1 capsid release into the cytosol.

## Experimental Procedures

### Virus Production and Purification

HSV-1 virions (wild-type strain 17^+^, kindly provided by Beate Sodeik, Hannover Medical School, Germany) were propagated in human foreskin fibroblasts (kindly provided by Prashant Desai, University of Baltimore, MD) and purified as described previously (Döhner et al., 2006; Maurer et al., 2008).

### Expression and Purification of the gB Ectodomain Constructs

The ectodomain lacking the MPR, viz. gB730t, residues 31–730, was expressed and purified from baculovirus as described previously (Bender et al., 2003; Cairns et al., 2011; Hannah et al., 2009). gB773t corresponds to the full-length ectodomain and included the MPR (residues 31–773). The gene fragment of gB773t was amplified using the following two primers: 5′-CGGCTGCAGTTTACGTACAA and 5′- CGCGAATTCAATTGGACATGAAGGAGGACAC and cloned into pCW289, generating pVT-Bac construct pLH633. Expression and purification of gB773t was as for gB730t (Cairns et al., 2011). SEC was done on a Superdex200 column (GE Healthcare), run with either 2× PBS or 2× PBS/0.5% octyl-glucoside (Calbiochem). To enable a quantitative comparison of the SEC runs with and without detergent for gB773t, the sample was split. One half was applied directly to SEC and run with 2× PBS. Detergent was added to the other half to provide a final concentration of 0.5% octyl-glucoside. After overnight incubation, the sample was applied to SEC and run in 2× PBS/0.5% octyl-glucoside.

### Negative Stain EM

Protein fractions, from a Superdex200 size exclusion column were applied onto carbon-coated glow-discharged EM grids at a concentration of 2–20 μg/ml. The grids were then stained with 0.75% uranyl formate, pH 5.5, or methylamine vanadate, pH 8.0 (Nanoprobes). The uranyl formate stain produced the better contrast in EM but is incompatible with neutral pH. The vanadate stain at pH 8.0 was used to rule out any pH-induced conformational changes induced by low pH. Microscopy was performed at 120 kV with a Tecnai T12 electron microscope (FEI), and images were recorded on a FEI Eagle camera.

### Liposome Flotation Assays and Dot Blots

Conditions for flotation experiments and dot blots were as previously described (Cairns et al., 2011; Hannah et al., 2009).

### Optimization of the Liposome-gB Interaction for Cryo-EM and Cryo-ET Data Collection

Liposomes consisting of phosphatidylcholine and cholesterol (Avanti Polar Lipids) at 1.7:1 molar ratio were prepared as previously described (Cairns et al., 2011; Hannah et al., 2009; Whitbeck et al., 2006). Protein samples of the trimeric fraction were concentrated to 2 mg/ml before they were mixed with (3–49 μg) freshly prepared liposomes in PBS at a pH of 5.5, 6.0, or 7.4 adjusted with sodium citrate. The final protein concentration in the mixture was ca. 1 mg/ml. The mixture was incubated for 1 hr at 37°C.

Samples with or without liposomes were applied onto glow-discharged holey carbon-coated EM grids (C-flat, Protochips). Colloidal gold particles with a diameter of 10 nm were added, and the grids were vitrified by plunge-freezing into liquid ethane.

Microscopy was performed at either 200 or 300 keV with a Tecnai F20 or a F30-Polara electron microscope (FEI) equipped with a GIF2002 postcolumn energy filter (Gatan) operated in zero loss mode. Images were recorded on either a 4k × 4k charge-coupled device (CCD; Ultrascan 4000, Gatan) at a calibrated magnification of 67,000, resulting in a pixel size of 0.23 nm or a 2k × 2k CCD at a calibrated magnification of 110,000, resulting in a pixel size of 0.27 nm at the specimen level. Projection images were recorded at 300 keV and defocus settings between −6 μm to −2 μm using SerialEM (Mastronarde, 2005). Tilt series were collected at 200 keV using SerialEM (Mastronarde, 2005) at a defocus of −4 μm or −2 μm in two-degree increments covering an angular range from −60° to 60°. The total electron dose for the tilt series was kept between 60 and 100 electrons/Å^2^.

### Tomographic Reconstructions and Subvolume Averaging

Tomographic reconstructions (nine in total, acquired at 200 keV) were calculated in IMOD (Kremer et al., 1996) using weighted back-projection (Sandberg et al., 2003). gB spikes (996 in total) in side view (664) and top view (332) orientations were manually picked from tomograms low-pass filtered to 1/8 nm spatial frequency.

The averaged structure was calculated by iterative alignment and averaging from unfiltered tomograms as implemented in the Jsubtomo package (Huiskonen et al., 2010). Iterative alignment and averaging was carried out in four stages (see below). The alignment and averaging process was iterated until convergence at each stage (Figure S4A). The initial model used for template matching was calculated by averaging all of the picked spikes.

Iterative alignment and averaging was carried out as follows: in the first stage, cylindrical symmetry was imposed on the averages and only the emerging angle from the membrane was refined by ±16° in 8° increments and allowed to shift by ten pixels. A large spherical mask (20 nm in diameter) was used. In the second stage, the angle around the spike was refined by ±180° in 8° increments. A tight cylindrical mask, only slightly larger than the spike, was used (height 18 nm, diameter 6 nm). The emerging angle from the membrane was kept fixed and no shifts were allowed. No symmetry was assumed nor imposed during this stage. Following the second stage, the cross-correlation coefficient was calculated for the map, and for the same map rotated in 1° increments around its long axis while applying the tight cylindrical mask. The cross-correlation coefficients were plotted as a function of the angle (Figure S4A). In the third stage, the angle around the spike was further refined by allowing it to change by ±60° in 8° increments and 3-fold symmetry was imposed on the average. Finally, at the fourth stage, all three angles were refined together to 8° accuracy. The angle around the spike was allowed to change ±60°, and the emerging angle from the membrane by ±16°. Maximum shifts of six pixels were allowed.

At each stage, the missing wedge was taken into account in calculating the constrained cross-correlation between the template and each of the subvolumes, by using a reciprocal space wedge-shaped mask reflecting the tilt geometry. The calculation was restricted also within a resolution band from 1/40 to 1/2.8 nm (to 1/2.3 nm in the final stage). The best correlating spikes (75%) from each liposome were included in the average. To exclude overlaps, particle locations closer than ten pixels were excluded. A total of 786 particles was used to calculate the final average. To map the lateral interactions between the spikes, the average was placed into the known orientations on the liposome membrane in two tomograms (Figures 5C and 5D).

### Pseudo-Atomic Modeling

The final cryo-ET density map (Figures 4C–4G) was segmented using Chimera (Pettersen et al., 2004; Pintilie et al., 2010) to a protein segment and a membrane segment. The gB crystal structures, viz at neutral and low pH (Protein Data Bank [PDB]: 2GUM and 3NWF, respectively; Heldwein et al., 2006; Stampfer et al., 2010) were initially rigidly fitted to the protein segment. The missing FL residues A261 and F262 in the low pH structure were modeled in all the three chains of gB using MODELER (Sali and Blundell, 1993) based on the gB crystal structure at neutral pH (PDB: 2GUM). A lipid bilayer was built using the Membrane Builder CHARMM-GUI (Jo et al., 2009) with a lipid composition like the experimental and fitted to the membrane segment.

The fitting into the protein segment was further improved by dividing the gB trimer into smaller rigid bodies, viz the crown region and middle were each a rigid body, and the base region was further divided based on clusters of secondary structure elements identified by RIBFIND (Pandurangan and Topf, 2012; Figure S5B). The Cα root-mean-square deviation (rmsd) between the initial fit of one rigid body and the fit with multiple rigid bodies was 3.42 Å and the component placement score (CPS; Pandurangan and Topf, 2012) for the base region of the gB revealed a translation of 2.71 Å and a rotation of 6° (Figure S5C). In a separate attempt, only the base multiple rigid bodies were fitted, which resulted in considerable conformational changes (Figure S5D). The conformation was then applied on a full chain followed by refinement with Flex-EM (Topf et al., 2008). Finally, 3-fold symmetry was applied. The Cα rmsd between the initial and the final fit was 40.36 Å. The translation and the rotation values calculated with CPS for the base domain were 4.17 Å and 8.48°, respectively. The global mutual information scores (Vasishtan and Topf, 2011) are 0.151 and 0.153, and the cross-correlations were 0.689 and 0.681 for the initial versus final fits, respectively. The local cross-correlation of the FLs was 0.184 for the acidic pH versus 0.210 for the neutral pH.

## Accession Numbers

The EM Data Bank accession numbers for the density map of the averaged gB-lipid bilayer map before and after refining the emerging angle from the membrane are EMD-2379 and EMD-2380, respectively. The Protein Data Bank accession number for the coordinates of the fitted model after refining the emerging angle from the membrane is 4BOM.

## Supplemental Information

Document S1. Supplemental Experimental Procedures, Figures S1–S5, and Table S1

Movie S1. Sequential Stages from the Initial Fitting of the Whole gB Trimer to the Final Pseudo-Atomic Model, Related to Figures 4H–4L
