# Supplementary material for: The Structure of Herpesvirus Fusion Glycoprotein B-Bilayer Complex Reveals the Protein-Membrane and Lateral Protein-Protein Interaction
Source: Structure. 2013 Aug 6;21(8):1396–405. doi: 10.1016/j.str.2013.05.018 (PMC3737472; doi:10.1016/j.str.2013.05.018)
Supplement: Document S1. Supplemental Experimental Procedures, Figures S1–S5, and Table S1 [file mmc1.pdf]

Structure, Volume *21*

Supplemental Information

The Structure of Herpesvirus Fusion Glycoprotein B

-Bilayer Complex Reveals the Protein-Membrane

and Lateral Protein-Protein Interaction

Ulrike E. Maurer, Tzviya Zeev-Ben-Mordehai, Arun Prasad Pandurangan, Tina M. Cairns, Brian P. Hannah, J. Charles Whitbeck, Roselyn J. Eisenberg, Gary H. Cohen, Maya Topf, Juha T. Huiskonen, and Kay Grünewald

<u>Inventory of Supplemental Information</u>

Figure S1, related to Figures 2-4

Figure S2, related to Figures 2

Figure S3, related to Figures 1 and 3.

Figure S4, related to Figure 4

Figure S5, related to Figure 4

Table S1, related to Figure 2

SUPPLEMENTAL INFORMATION

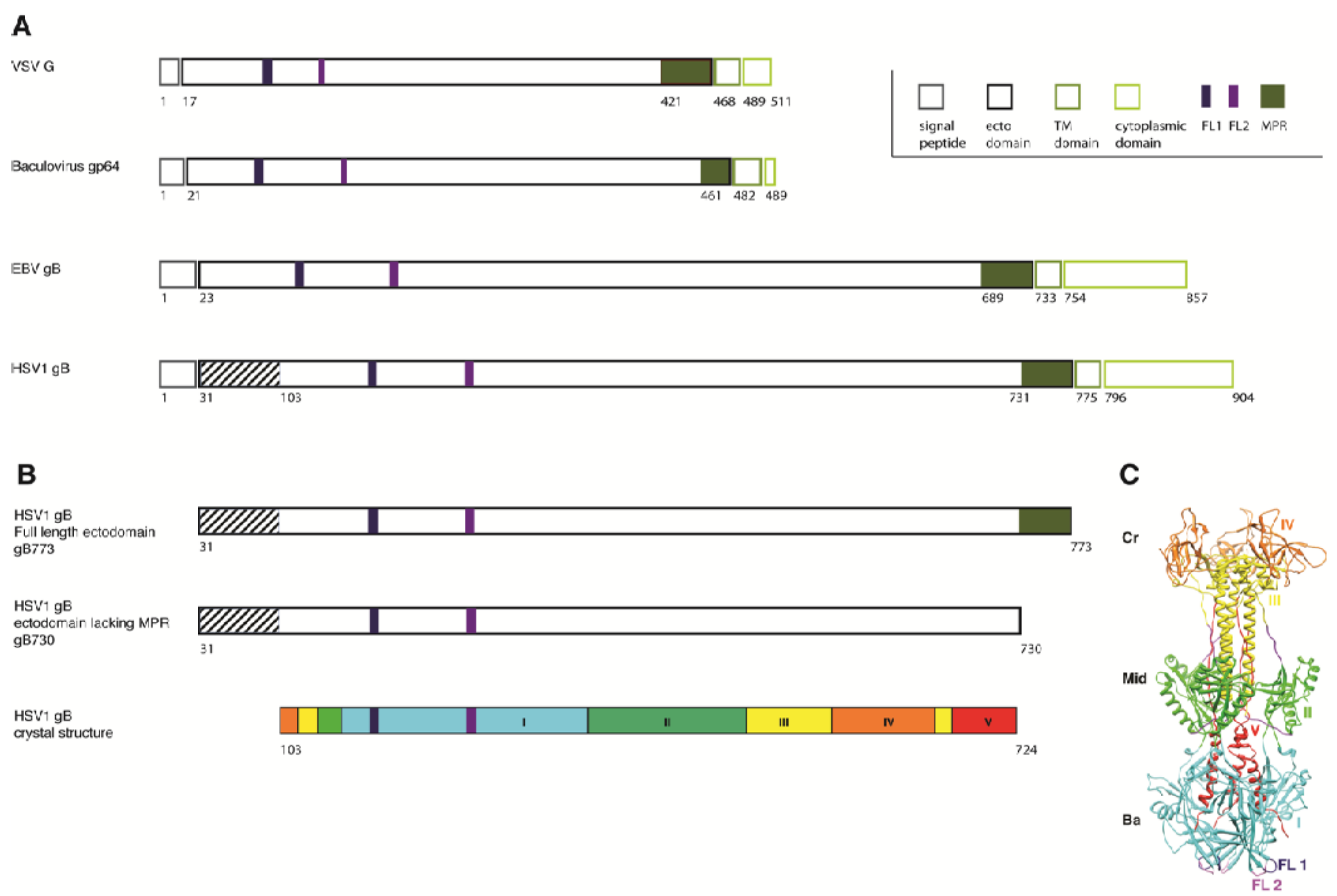

Figure S1, related to Figures 2-4. Schematic representation of (A) the general topology of members of the class III fusion proteins, as previously assigned (Backovic et al., 2007; Backovic et al., 2009; Kadlec et al., 2008; Roche et al., 2006) and (B) the different HSV1 gB ectodomain constructs referred to here. All class III fusion proteins differ in their ectodomain length but commonly have a hydrophobic membrane proximal region (MPR) at the ectodomain C-terminus. The ectodomain of HSV1 gB additionally contains an unassigned 70-amino-acid long region (diagonally hatched) on its N-terminus that is conserved in alphaherpesviruses. All crystal structures of class III fusogens have been determined for constructs lacking the MPR. The crystal structures of HSV1 gB additionally lack the N-terminus unassigned domain. The assigned domains based on the crystal structure, (C) (Stampfer et al., 2010) are colored: the base domain (I, cyan), middle domain (II, green), core domain (III, yellow), crown domain (IV, orange) and arm (V, red). TM, transmembrane; FL, fusion loop.

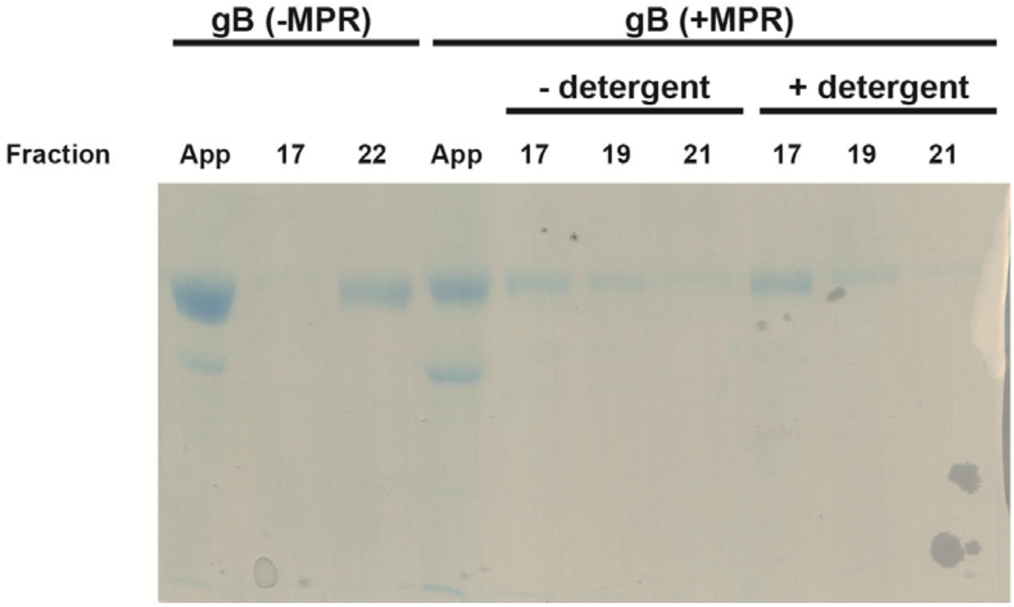

Figure S2, related to Figure 2B. SDS-PAGE of size exclusion chromatography fractions (App, sample applied to SEC).

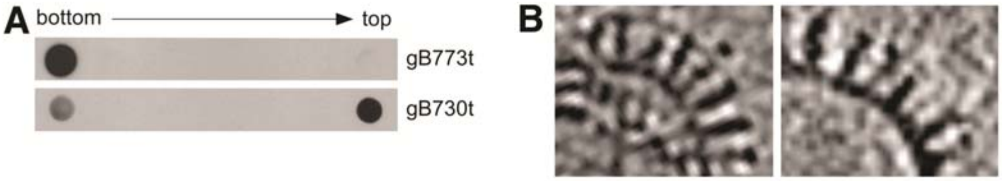

Figure S3, related to Figures 1 and 3. (A) Dot blots of fractions from co-flotation assay probed with anti-gB antibody. gB730t, ectodomain lacking the MPR; gB773t, full-length ectodomain including the MPR. Liposome-associated proteins float to the top fractions, while non-associated proteins remain at the bottom.
(B) Comparison of clustering of gB (left) on native HSV1 virions (from Figure 1 inset) and (right) ectodomains lacking the MPR on liposomes (*cf*. Figure 3H).

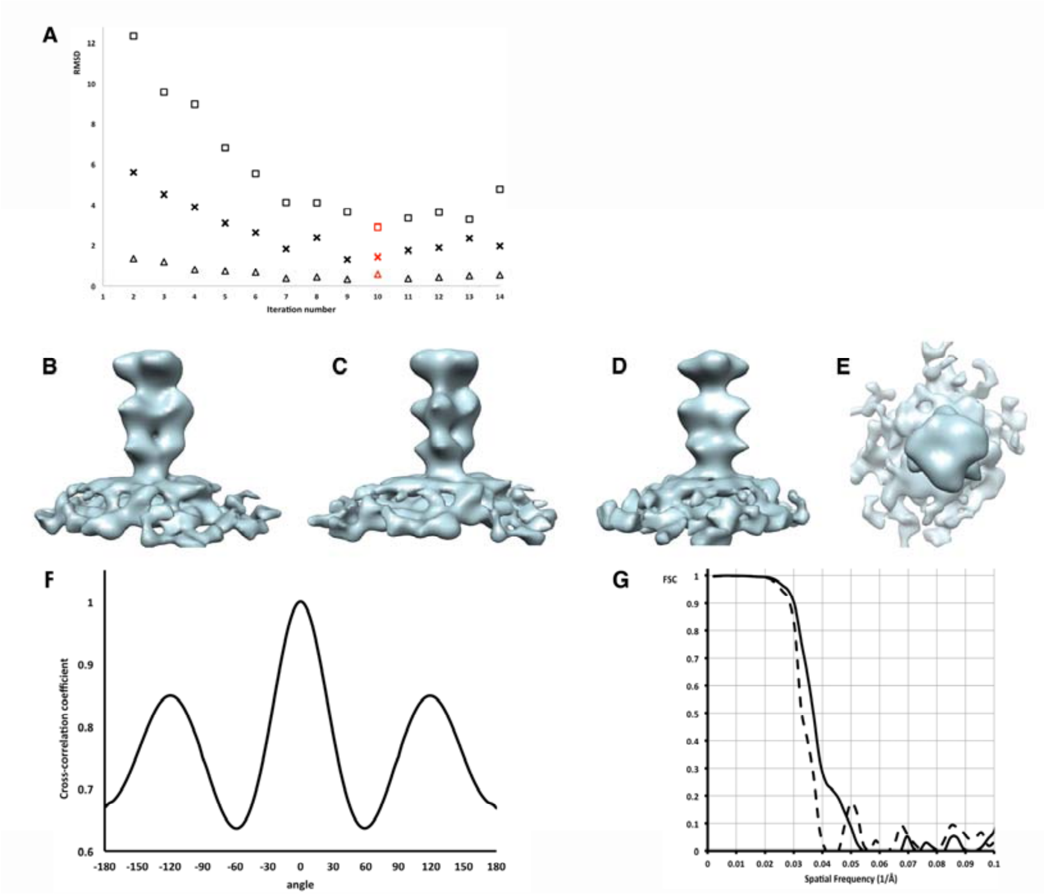

Figure S4, related to Figure 4 Subtomogram averaging of gB membrane complex (A) Convergence of the iterative alignment and averaging process in the final stage of refinement. RMSD between each iteration and the previous one is plotted for the angle around the view vector (open square, in degrees), direction of the view vector (cross, in degrees) and the particle location (open triangle, in pixels). The final map shown in Figure 4C-L was from iteration 10 (marked in red). (B-F) Three-fold symmetry was apparent in the averaged density early in the iterative refinement process. (B-E) Isosurface rendering of the unsymmetrised average. Views rotated sequentially by 30° around the long axis of the protein are shown in (B-D) and an orthogonal view to (D) onto the crown region is shown in (E). (F) Rotational self-correlation plot of the average. (G) Resolution assessment of the averages. Fourier shell correlation is plotted for the average in which the emerging angle of the spikes were assumed to be normal to the membrane and kept fixed during refinement (dashed line; see also Figure 4A and B) and for the final refined average after emerging angle refinement (solid line; see also Figure 4C-L).

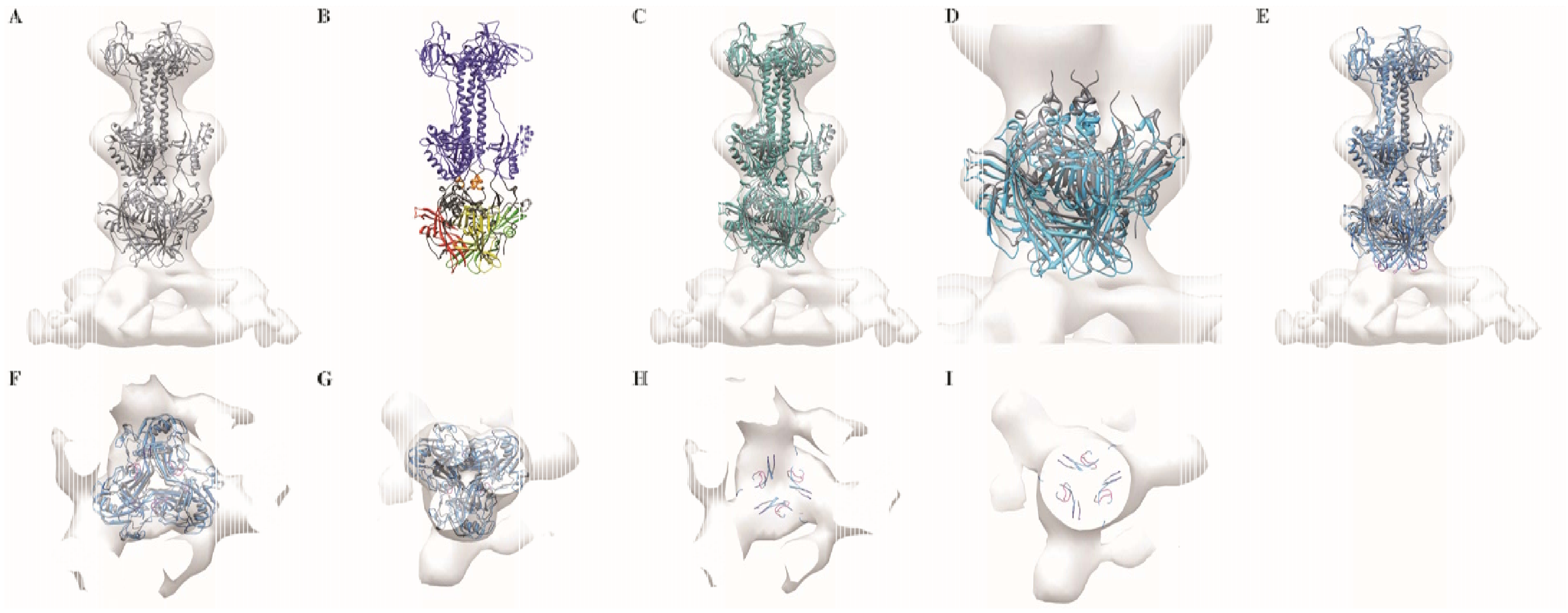

Figure S5, related to Figure 4. Comparison of sequential stages of flexible fitting to the initial fitting of the whole gB trimer (shown in ribbon representation). (A) Initial fitting of the whole gB trimer crystal structure (PDB: 3nwf; grey) into the final cryoET density map (Figure 4C-G). (B) Clusters of secondary structure elements identified by RIBFIND (Pandurangan and Topf, 2012) for the base region and the three small helices linking it to the middle region, coloured respectively red, yellow, green and orange. Unclustered elements are coloured dark grey. The crown and the middle regions are coloured blue. Each coloured cluster was treated as a single rigid body during the subsequent flexible fitting. (C) Superposition of the initial fit (grey) and the flexible fit (sky blue) of the identified clusters shown in (B). (D) Superposition of the initial fit (grey) and the flexible fit (sky blue) applied to the base domain of gB only. (E) Superposition of the initial fit (grey) and the final fit (dodger blue) of gB (as shown in Figure 4H-L). (F) The base region from the superpositioned fits presented in (E) rotated 90º about the horizontal axis and viewed from the membrane side or (G) viewed from the protein side. (H) Thin slice at the protein-membrane interface of the superpositioned fits presented in (E) rotated 90º about the horizontal axis and viewed from the membrane side or (I) viewed from the protein side. Fusion loops 1 and 2 are coloured magenta and dark blue, respectively.

Table S1, related to Figure 2

Hydrophobicity of residues forming the putative fusion loops and the membrane proximal regions (MPR) of members of the class III fusion proteins.

**Protein name**[a]	**Fusion Loop 1**			**Fusion Loop 2**			**MPR**		
	Residues[b]	KD[c]	CCS[d]	Residues[b]	KD[c]	CCS[d]	Residues[b]	KD[c]	CCS[d]
**VSV G**	DFRWYGPK[85-92]	-1.66	-1.07	GYATV[131-135]	0.72	-0.14	421-467	-0.42	-0.7
**Baculo gp64**	GGSLDPNT[79-85]	-0.88	-2.35	NNNHFA[149-154]	-1.51	-2.7	461-481	0.36	-0.98
**EBV gB**	GWYA[111–114]	-0.2	2.17	WLIWT[193–197]	1.15	6.8	689-729	0.94	1.09
**HSV1 gB**	GHRY[176–179]	-2.35	-3.42	RVEAF[258–262]	0.16	-1.06	731-773	1.18	0.81

[a]Protein names and corresponding Swiss–Prot entry number: glycoprotein G of the vesicular stomatitis virus (VSV G, P03522), baculovirus major envelope glycoprotein gp64 (baculo gp64, P17501), and Glycoprotein B of the herpes simplex 1 (HSV1 gB, P06437) and Epstein–Barr virus (EBV gB, P03188).

[b]Numbering of residues corresponds to that of unprocessed proteins, taken from original publications.

[c]Average hydrophobicity calculated based on Kyte and Doolittle (KD) scale (Montgomery et al., 1996).

[d]Average hydrophobicity calculated based on combined consensus scale (CCS) (Leikina et al., 2004). The larger number corresponds to higher hydrophobicity.

SUPPLEMENTAL REFERENCES

Backovic, M., Leser, G.P., Lamb, R.A., Longnecker, R., and Jardetzky, T.S. (2007). Characterization of EBV gB indicates properties of both class I and class II viral fusion proteins. Virology *368*, 102-113.

Backovic, M., Longnecker, R., and Jardetzky, T.S. (2009). Structure of a trimeric variant of the Epstein-Barr virus glycoprotein B. Proc Natl Acad Sci U S A *106*, 2880-2885.

Kadlec, J., Loureiro, S., Abrescia, N.G., Stuart, D.I., and Jones, I.M. (2008). The postfusion structure of baculovirus gp64 supports a unified view of viral fusion machines. Nat Struct Mol Biol *15*, 1024-1030.

Leikina, E., Mittal, A., Cho, M.S., Melikov, K., Kozlov, M.M., and Chernomordik, L.V. (2004). Influenza hemagglutinins outside of the contact zone are necessary for fusion pore expansion. The Journal of biological chemistry *279*, 26526-26532.

Montgomery, R.I., Warner, M.S., Lum, B.J., and Spear, P.G. (1996). Herpes simplex virus-1 entry into cells mediated by a novel member of the TNF/NGF receptor family. Cell *87*, 427-436.

Pandurangan, A.P., and Topf, M. (2012). Finding rigid bodies in protein structures: Application to flexible fitting into cryoEM maps. Journal of structural biology *177*, 520-531.

Roche, S., Bressanelli, S., Rey, F.A., and Gaudin, Y. (2006). Crystal structure of the low-pH form of the vesicular stomatitis virus glycoprotein G. Science *313*, 187-191.

Stampfer, S.D., Lou, H., Cohen, G.H., Eisenberg, R.J., and Heldwein, E.E. (2010). Structural basis of local, pH-dependent conformational changes in glycoprotein B from herpes simplex virus type 1. J Virol *84*, 12924-12933.
